# Utilizing the Walla Emotion Model to Standardize Terminological Clarity for AI-Driven “Emotion” Recognition

**DOI:** 10.3390/brainsci16030260

**Published:** 2026-02-26

**Authors:** Peter Walla

**Affiliations:** 1 Freud CanBeLab, Faculty of Psychology, Sigmund Freud Private University, Freudplatz 1, 1020 Vienna, Austria; peter.walla@sfu.ac.at; 2 Faculty of Medicine, Sigmund Freud Private University, Freudplatz 3, 1020 Vienna, Austria; 3 School of Psychology, Newcastle University, University Drive, Callaghan, NSW 2308, Australia

**Keywords:** affective computing, affective processing, cognitive processing, feeling, emotion, mood, affect, motivation, non-conscious processing, consciousness, emotion recognition, algorithms, AI, artificial intelligence

## Abstract

The scientific study of affect has been historically characterized by a profound lack of terminological consensus, leading to a state of conceptual fragmentation that persists in psychology, neuroscience and many other fields. This ambiguity is not merely an academic concern; it has significant consequences for the development of artificial intelligence (AI) systems designed to recognize and respond to human “emotions”. In fact, it has an influence on the entire field of affective computing. The problem is obvious. Without a distinct definition of “emotion” it is difficult to train an algorithm to recognize it. The Walla Emotion Model, also known as the ESCAPE (Emotions Convey Affective Processing Effects) model, provides a potentially helpful and neurobiologically grounded framework to resolve this impasse and to improve any discourse about it, for businesses and even lawmakers aiming at healthy societies. By establishing clear, non-overlapping definitions for affective processing, feelings, and emotions, this model offers a path toward more precise research and more ethically sound affective computing including AI-driven “emotion” recognition. It introduces a concept that allows for the detection of incongruences between internal states and external signals with a very clear terminology supporting understandable communication. This is critical for identifying feigned or socially masked inner affective states, a challenge that traditional “face-reading” AI models frequently fail to address. Even tone of voice and body postures as well as gestures can be and are often voluntarily modified. Through the separation of subcortical affective processing (evaluation of valence; neural activity) from subjective experience (feeling) and external communication (emotion), the Walla model provides a helpful framework for AI-designs meant to have the capacity to infer an internal affective state from collected signals in the wild bypassing verbal self-report. This paper is purely theoretical; it does not provide any algorithm models or other distinct suggestions to train a software package. Its main purpose is the introduction of a new emotion model, particularly a new terminology that is considered helpful in order to proceed with this endeavor. It is considered important to first enable the clearest-possible form of communication about anything related to the term emotion across all disciplines dealing with it. Only then can progress be made.

## 1. Introduction

The essence of this essay and probably the best argument for it is a very trivial question. How shall an algorithm know what to recognize, when even humans do not? For over a century, the term “emotion” has been utilized as a comprehensive designation for a disparate set of phenomena, including physiological responses, conscious experiences, cognitive appraisals, and behavioral expressions. Modern AI systems are meant to be trained to recognize human emotions, but how shall this endeavor be a solid and serious goal, when nobody knows what an emotion actually is? This theoretical paper aims to clarify this problem by introducing a new emotion model. This model mainly proposes a new terminology that clearly separates and specifically defines the terms “affective processing” (affection), “feeling” and “emotion”. However, before explaining these neurobiologically rooted concepts in more detail, a short description of other existing models is provided in the following section.

The landscape of emotion research is characterized by a fundamental tension between “nature” (biological essentialism) and “nurture” (cognitive construction). Early foundational models emphasized the evolutionary and biological origins of “emotion”. Plutchik [1] and Ekman [2,3] proposed that a limited set of “basic emotions” are hardwired, universal, and evolved for survival. Ekman’s work on facial expressions remains the bedrock for modern automated emotion recognition, though it is increasingly scrutinized for its reliance on outward social signals. Similarly, Izard [4] highlights the distinct functions of these primary systems in human development. Moving toward the brain’s hardware, Panksepp [5,6] established the field of affective neuroscience, identifying subcortical “core emotional feelings” shared across mammals. LeDoux [7] further refined this by rethinking the “emotional brain,” distinguishing between survival circuits (like fear) and the conscious experience of an emotion. Damasio [8,9,10] revolutionized the field with his “Somatic Marker Hypothesis,” arguing that emotions are not just “mental”, but are rooted in bodily states (“the feeling of what happens”), which are essential for rational decision-making. In contrast to purely biological views, Lazarus [11] and Ortony, Clore, and Collins [12] introduced complex appraisal models. They argue that emotions arise from the cognitive evaluation of how an event impacts personal goals (e.g., the OCC model). Rolls [13,14] complements this by defining emotions as states elicited by reinforcers (rewards and punishers), providing a functional framework for how motivation and reasoning are linked to neural systems. Cabanac [15] supports this by viewing emotion as a “common currency” for survival-based trade-offs. Challenging the idea of hardwired fingerprints for emotion, Russell [16] proposed the Circumplex Model, where affect is mapped alongside the dimensions of valence (pleasurableness) and arousal. This dimensional approach was significantly expanded by Barrett [17,18]. Her “Theory of Constructed Emotion” posits that emotions are not triggered, but are active inferences constructed by the brain based on interoception and past experience. This solves the “emotion paradox”, the discrepancy between our subjective feeling of distinct emotions and the lack of distinct physiological markers for them. Finally, Scherer [19] proposed the Component Process Model (CPM), which views emotion as a highly synchronized, dynamic process involving multiple components, which are appraisal, bodily symptoms, action tendencies, and motor expressions.

While the above list does not even include all existing schools of science on “emotion”, it becomes evident enough that there is no common understanding of how to define “emotion”. Thus, how should an AI designer know what to train an algorithm for? While traditional AI often relies on the discrete models of Ekman, modern research increasingly draws on the neurobiological insights of Damasio and the constructivist critiques of Barrett. This shift highlights a critical gap. Traditional AI measures the social “output” (expression), whereas the “biological truth” lies in the underlying “Action Program” (affective processing; the raw data). By understanding the history of these models, one can better appreciate why measuring non-conscious physiological responses representing affective information processing is essential to bypass the “social mask” and capture the true internal affective state that represents the main driver for decision-making and finally for produced human behavior. The broad and inconsistent usage of terminology [20] has led to a theoretical quagmire where researchers often measure different aspects of affective responses while using the same vocabulary, resulting in data that are difficult to compare and synthesize. Since communication is the key to everything, a clear solution is highly desired. Traditional models often assume a direct, universal, and involuntary link between internal states and facial expressions; an assumption that has been increasingly discredited by evidence of cultural diversity and individual variability as well as the capacity to voluntarily generate fake facial expressions and also gestures, even tone of voice.

McStay (2018) [21] documents the rapid shift toward “Empathic AI”: systems designed to sense, learn, and react to human “emotional states”. He highlights how the integration of computer vision and machine learning into everyday devices (from smartphones to cars) has turned “emotion” recognition into a multibillion-dollar industry and a central focus of modern AI research [21]. However, at the same time, when taking a closer look at the bulk of the literature on the “emotion” topic, it is hard to understand why such a big deal is made on the basis of unclear understandings of the key essence, an emotion. Much like how an AI trainer does not really know on what basis they should train an algorithm, other professionals also struggle. Imagine your therapist, with good intentions, suggesting you practice emotional regulation. Currently, you would not know what to regulate and different therapists would explain the workings of emotional regulation in different ways.

Only recently has the already mentioned approach to handle the inconsistently used vocabulary around “emotion” been suggested. Under the title “A call for conceptual clarity: “Emotion” as an umbrella term did not work—let’s narrow it down” [22], Walla et al. propose an alternative terminology that is meant to improve any communication, be it psychological, philosophical, or clinical, with respect to AI and even for lawmakers that increasingly deal with AI-driven emotion recognition. In contrast to the above-mentioned existing emotion theories, the Walla Emotion Model, introduced here, provides a clear set of three definitions relevant to the AI domain (affective processing, feeling, emotion) in addition to various other disciplines dealing with “emotions”. Interestingly, recently published AI-related work began to avoid using the term emotion at all in the context of AI [23,24]. This may sound like a good alternative. However, given the strong connections between scientific disciplines that have been dealing with “emotions” for almost centuries (philosophy, psychology, therapy, biology, medicine, etc.) and the AI domain, I consider it more helpful to use consistent terminology across all those fields while still including the term emotion, but with a different and more precise set of definitions for all the relevant phenomena related to it. Surely, psychology and therapy, for instance, will not give up using the term emotion, and thus it might be more helpful to give it a more precise definition, in order to separate it from feelings, affective processing and also from cognition rather than avoiding it. In the following sections, this new and potentially helpful emotion model is explained, including a table summarizing its main three concepts (affective processing, feeling and emotion), while also emphasizing its benefits for AI.

## 2. The Evolution of the Walla Emotion Model

The Walla Emotion Model [22] emerges from a critical appraisal of historical failures, particularly the interchangeable use of “affection (or affective processing)”, “feeling,” and “emotion” [25,26], but also from a lack of separating affection from cognition. It builds upon the insights of early pioneers like Charles Darwin [27], who focused on the communicative function of muscle contractions, and William James [28], who emphasized the link between bodily changes and felt experience. Walla et al. [22] introduce a crucial distinction by separating the processing of information (i.e., affective processing; the raw data) from the experience related to that information due to its bodily consequences (i.e., feeling; subjective experience) and the signaling related to it to the social environment (i.e., emotion; behavioral output as a result of muscle contractions). This separation is grounded in the hierarchical evolution of the brain. The human brain operates as a multidimensional system where different layers of processing developed at different evolutionary stages, but function together as a unified whole [29]. Subcortical structures, which are evolutionarily older, perform rapid, survival-oriented evaluations of the environment long before the cortical regions, which are responsible for language and reasoning, become involved [30]. The Walla model aligns its definitions with these biological realities, prioritizing the objective measurement of neural activity over the subjective interpretation of labels when it comes to better understanding how humans respond with affection to any stimulation (external and internal). Affection represents the very basis for decision-making, which means that getting access to it offers the best possible insight into human behavior influence and even its prediction. A detailed explanation including neurobiological and evolutionary backgrounds can be found in the original article [22], but in the following section, you will find a short list of the already mentioned three main components of this model that are relevant to the AI field:

- Affective Processing: The rapid, unconscious neural evaluation of stimuli based on valence (pleasantness/unpleasantness) and arousal (intensity). This occurs primarily in the limbic system and guides initial behavioral tendencies like approach or avoidance. This is in fact the most crucial raw data that underlies feelings and emotions.
- Feelings: The conscious, subjective experiences that arise when affective processing exceeds a specific threshold, triggering the release of neurochemicals that alter the internal bodily state. These are the “felt” internal responses, such as a knot in the stomach or a sense of unease. This is the level that forms the basis for verbal reports on how one feels.
- Emotions: Strictly defined as the external behavioral outputs—facial expressions, vocalizations, and body postures as well as gestures—that serve to communicate a felt state to others. Interestingly, there are automatic involuntary emotions, but there is also the possibility to generate voluntary emotions. This might be most critical to AI-designs, because of potential masking.

Affective processing is the most fundamental level of brain function (the subcortical evaluative core; the raw affective data source). It represents the “brain automatically evaluating” something is good or bad, safe or dangerous, before the conscious “I” realizes it. This process is continuous and automatic, providing a constant evaluative stream that informs behavioral adaptation and guides human behavior most dominantly [25,26]. Affective processing is mapped onto two primary dimensions: valence and arousal. Valence refers to the motivational direction: whether the brain evaluates a stimulus as something to be approached (positive) or avoided (negative). Arousal denotes the intensity of the evaluation or the degree of physiological activation. Both valence- and arousal-related neural data feed (as action tendencies) into decision-making centers that plan the execution of adapted behavior. The limbic system, including the amygdala, hippocampus, and hypothalamus, is the primary seat of this processing level [31]. These structures receive sensory input and perform a rapid assessment based on evolutionary and learned associations. Because this processing happens deeply subcortically, it is not directly accessible to the cortical functions of language and conscious reflection. This leads to the phenomenon of “gut reactions”: responses that a person feels strongly, but cannot immediately explain or justify verbally. It also leads to cognitive pollution, which is dealt with further below. Whatever happens on this level of information processing represents the true affective reaction of a human being. This level represents the most interesting processing quality that one would want to get access to in order to best possibly understand human behavior. To demonstrate an example, a reliable method, also explained further below, to capture reactions of this level of information processing revealed that depressed people respond positively via verbal report to positive image presentations, while their measured raw affection reflects significantly more negative image evaluations deep inside the brain compared to healthy controls [32]. In other words, getting access to the raw affection level provides a quantifiable view of depression that does not show up via self-report. Furthermore, the brain of a psychopath processes disgusting images like accident victims or even images of mutilated bodies significantly more positively, although the verbal report level is similar to healthy controls [33].

To understand the Walla Emotion Model in the context of AI, it is crucial to recognize its foundational departure from traditional “Discrete Emotion” theories (like Ekman’s). While most AI models today are trained to label a face as “happy” or “sad,” the Walla model argues that these are merely social outputs that often mask the true biological state (i.e., raw affection). Below, we will discuss how modern AI systems (several companies) started to amend sole standard facial expression data as suggested by the well-known FACS (Facial Action Coding System) [34], using data such as tone of voice, body gestures and micro-expressions, but first, the following table (Table 1) breaks down the model into its three distinct phenomena and explains why this structured view could become an important basis for the next generation of AI that is meant to recognize “emotions”.

Current AI-driven emotion recognition often suffers from high error rates, because it assumes the face is a direct window to the soul. Humans often “think” about how they should feel, which pollutes self-reports and facial expressions. Focusing on affective processing is the goal, because this level of information processing is immune to this pollution. For example, by comparing FACS information with physiological data that directly represents raw affection, AI could be able to mathematically calculate the degree of social masking by identifying a mismatch between raw affection and the way a person talks about a certain topic or their facial expression. Even though some companies offer a multi-model approach, the masking problem remains. However, this theoretical paper does not aim at evaluating any AI packages that are available for emotion recognition; instead, it is meant to improve communication about them.

## 3. The Independence of Affection and Cognition

A key postulate of the Walla model is that affective processing does primarily occur independent of, and precedes, cognitive processing. While cognition asks “What is this?” (semantic identification), affection asks “How is this?” (evaluative significance). In terms of evolution, the ability to rapidly evaluate threats was essential for survival long before the development of complex language or abstract reasoning to answer “What-questions” [26]. This independence has significant implications for how we understand decision-making. Even in domains traditionally viewed as purely rational, such as financial asset management, subcortical affective processing plays a decisive role [35]. Human brains integrate affective “how” information with cognitive “what” information to navigate through complex environments, but the two streams are initially processed by distinct and separate neural pathways. Consequently, any emotion theory including cognitive aspects is considered potentially misleading according to the model introduced here.

### 3.1. Feelings and the Barrier of Conscious Reflection

According to the Walla model, feelings are defined as the subjective, conscious perception of the physiological changes triggered by intense affective processing. When neural activity in the limbic system reaches a certain threshold, it stimulates the release of neurotransmitters and hormones. These chemicals alter the body’s internal state, and the brain’s conscious monitoring of these changes is what we experience as a feeling. However, when asked to verbalize a feeling, one can certainly try, but might fail, because affective processing causing feelings was never meant to be verbalized anyway due to its evolution way before language came into existence. This led to the notion of cognitive pollution [26]. Clearly, people do not always do what they say.

### 3.2. The Phenomenon of Cognitive Pollution

Because affective processing refers to subcortical processing, and language is a cortical, cognitive function [36], the act of verbalizing affective content inevitably distorts the original data. It is similarly difficult with respect to feelings, even though they are conscious experiences. Actually, perhaps because they are conscious experiences like all perceptions are, they might be prone to mistakes similar to optical illusions. When an individual is asked, “How do you feel?”, they must engage higher-order reasoning to translate an abstract internal state into concrete words. This process of translation introduces several layers of bias, including social desirability, cultural expectations, and the limitations of the individual’s vocabulary. In the end, any conscious perception is nothing more than a construct of our psyche (not a one-to-one representation of initial stimulation). A resulting self-report is a “polluted” version of the raw affective state: a cognitive reflection rather than an accurate measurement of the underlying processing. This is why traditional research relying on surveys often finds discrepancies between what people say they feel and how their bodies or brains actually respond. In empirical research, this often leads to discrepancies between implicit and explicit measures.

### 3.3. Discrepancies Between Explicit and Implicit Measures

The new model advocates for the use of objective, implicit measures to capture “raw” affection, as these bypass the pollution of conscious reflection. Studies in neuromarketing, for instance, have shown that while participants might explicitly rate two brands similarly in a survey, their physiological responses (e.g., measured heart rate, skin conductance (SC), startle reflex modulation (SRM), electroencephalogram) reveal a clear preference for one over the other [37]. See Table 2 for a short summary of implicit and explicit measures.

## 4. Emotion as Communication

For AI, the Walla model’s most distinctive alternative is the reclassification of an “emotion” as a behavioral output rather than an internal state. Etymologically derived from the Latin verb “emovere” (to move out), an emotion is literally the “out-movement” of an internal state into the social world. This includes facial expressions, changes in vocal tone, gestures, body postures and many more behaviors. From an evolutionary perspective, the function of an emotion is social communication. By expressing an inner state like “fear” (i.e., affective processing causing the feeling of fear), an individual signals a perceived threat to their conspecifics, facilitating group survival. This signal is intended to convey a feeling, but the model emphasizes that the signal (the emotion) and the source (the feeling or affective processing) are not always functionally connected, but can be distinct for intentional nonverbal communication (e.g., fake smile). Perhaps this is the most important point in the context of AI. Following this model, one can say that AI is certainly capable of recognizing an emotion (behavioral output), but can it successfully interpret underlying affective processing level activity (raw affection)? Rather than arguing about the question of whether AI will ever be able to recognize human emotions without knowing what they actually are, the terminology introduced here at least allows for a meaningful discussion about what can be done already and what still needs improvement.

**Figure 1:**
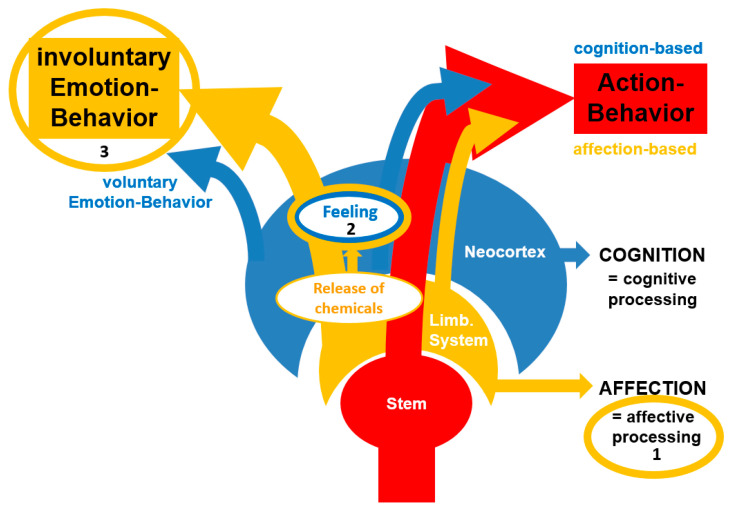
(1) Affective processing guides behavior by providing evaluative information to support decision-making for the production of action-behavior. (2) High levels of affective processing activity lead to released chemicals, which is felt by an organism that has consciousness. (3) Emotion-behavior is produced to communicate a felt state to conspecifics (involuntary and voluntary). This figure is taken from Walla et al., (2025) [22].

In the context of the already mentioned masking problem, this model allows for the existence of both involuntary and voluntary emotions. While some expressions are produced automatically as a result of above-threshold affective processing (e.g., a genuine scream of terror), humans have also developed the cortical (cognitive) capacity to produce voluntary emotions to feign a state that they do not actually feel. This is common in social “masking” or strategic emotional management, where individuals display a “happy face” to maintain social harmony despite feeling internal distress. The figure below (Figure 1) represents a visualization of this model, which distinguishes between action-behavior and emotion-behavior.

## 5. The “Diagnostic Gap” for AI Recognition

The functional separation of emotions from the internal state is the foundation of the “diagnostic gap”: the potential for a discrepancy between what is shown and what is felt. For AI-driven emotion recognition, this gap is the primary source of error. Most current systems are trained on the assumption that a “happy face” is happiness. The Walla model provides a scientific argument for why this is potentially flawed. The face is merely a signal that may or may not be congruent with the internal state. To be truly helpful, an AI model should be able to recognize such an incongruence. If an AI model detects a smile (emotion), but for example simultaneously measures low heart rate or low skin conductance (affective processing markers), it can infer that the smile is a social performance rather than a reflection of a genuine felt state. The practical implementation of this requires moving away from unimodal facial recognition toward multimodal triangulation. This involves measuring behavioral output alongside objective markers of raw affective processing.

Some of the most prominent technologies in the field of affective computing already address the problem of emotion masking. Although they do not really have built-in features to detect emotion masking, their developers seem aware of the problem. An important aspect of such software is that it is sensitive to micro-expressions, which are understood as involuntary emotions (according to Walla, 2025 [22]) more directly reflective of an internal state than more strong facial expressions that are more likely to be fake. Some software packages use datasets to classify expressions into “complex emotions” like joy, sadness, anger, surprise and contempt. The problem with that is that their often huge databases consist of face images and videos. By calling such data “emotions”, while at the same time understanding “emotion recognition” not primarily as recognizing facial expressions, but rather as interpreting inner affective states that generate the respective facial expressions, the problem becomes evident. Terminology around “emotion” is used interchangeably, causing confusion, inconsistency and inaccurate communication and, in the worst case, inaccurate inner state interpretations.

Other available software recognizes the limitations of relying solely on facial expressions and is actively moving toward a multimodal approach to capture a more “truthful” affective picture. While they do not use the term “emotion masking” as a primary product feature, their recent technological shifts are designed to at least try to solve the very problem that potential masking creates. At the moment, a core strategy for addressing the incompleteness of facial signals is the integration of speech and tone of voice. Respective designers acknowledge that facial expressions are only one channel of communication. By adding acoustic analysis including measures like pitch, tempo, and pauses, they attempt to cross-validate what the face is showing. If a face shows a “polite smile” (masked), but the voice remains flat or shows signs of “filler words” (um, uh) indicating cognitive load, the system can provide a more nuanced report. Furthermore, the software is designed to catch micro-expressions, which are, as mentioned above, more directly reflective of a true inner state.

Currently, perhaps the most reliable method to capture true and raw affection is Startle Reflex Modulation (SRM) [38,39,40,41]. Although SRM would currently be far away from being used in the current context of AI-driven emotion recognition, the following paragraph introduces this method, which is considered superior to all brain imaging tools regarding its capacity to quantify raw affection.

### Startle Reflex Modulation (SRM) as a “Gold Standard”

SRM involves measuring the amplitude of the eye-blink reflex in response to a sudden, loud sound (an acoustic startle probe). This reflex is controlled by the brainstem and is extremely difficult to voluntarily suppress. Crucially, the magnitude of the blink is modulated by the person’s current affective inner state [39,40]. If a person is in a positive state (e.g., viewing an image their affective system evaluates as positive), the blink magnitude is reduced. If they are in a negative state (e.g., viewing an image their affective system evaluates as negative), the blink magnitude is increased. Because SRM happens so quickly (within milliseconds) and is governed by subcortical pathways, it provides a “raw” measure of valence evaluation that is immune to cognitive pollution. The above-mentioned two studies, one about depression and the other on psychopaths, were done by utilizing SRM. While SRM is considered the gold standard to measure raw affective responses, it currently still requires a laboratory setup. However, from a theoretical perspective, by synthesizing data from multiple channels, researchers (and also AI) can create a comprehensive profile including all levels starting with the raw affective data, feelings and expressive output (emotions). Maybe, the future will bring wearable devices or other techniques to use the concept of SRM. At this stage, most importantly, it has to be accepted that the sole interpretation of behavioral data will not be sufficient to decode a person’s inner state of affect.

## 6. Beyond Pixel-Level Accuracy

Current AI systems for emotion recognition are often limited by “ground truth” labels that are fundamentally flawed [42,43]. Most datasets are labeled by humans, who look at a face and guess the underlying affective state. If the person in the image is faking their expression, the AI is trained to recognize the fake expression as the “truth”. AI needs to shift its focus from “accuracy” (matching a human label) to “veracity” (matching the subcortical state). A further problem is potential anthropomorphism and simulated affect.

A significant challenge in current AI (especially Large Language Models) is the simulation of empathy [44,45,46]. LLMs can produce text that “feels” affectively aware, because they have learned the patterns of affective language from massive datasets. However, as the Walla model highlights, these systems lack the subcortical machinery of affective processing. They are producing “emotions” (behavioral output) without “feelings” (conscious experience) and underlying “affective processing” (biological evaluation). This can lead to “affective delusion,” where users believe the AI “really likes” them, but during following conversations the opposite can result in nonbiological, or at least strange, feelings arising in a user [46,47,48]. There are even cases of suicide in response to seemingly empathic AI [49]. AI is essentially a “stochastic parrot” of social signals: it can mimic the output, but it cannot experience the source. While this potentially creates a wanted response in the person exposed to (or confronted by) the AI model, it might be a better alternative to let the person know about the actual fakeness rather than allowing a piece of software to feign real affection. For AI developers, this insight is crucial for setting realistic boundaries and preventing “persona drift” or harmful affective manipulation in human–machine interactions.

## 7. Ethical and Regulatory Implications

As AI-driven emotion recognition moves from the laboratory into the public sphere, the emotion model proposed here may provide a helpful framework for addressing ethical concerns and regulatory requirements, such as the problem of affective surveillance. If an emotion is defined as a behavioral signal, then tracking it is akin to tracking a person’s public speech or body language. However, if one claims to be “reading feelings” or even raw affective processing, one is asserting the right to peer into a person’s most private internal states. The Walla model helps draw a clear line. Most current AI is only recognizing emotions (the signal), not feelings (the experience), nor raw affective responses. Failure to make this distinction leads to a “pseudoscience” of affective surveillance, where authorities believe they can unearth hidden “intentions” or “guilt” from a facial expression. Regulatory frameworks, such as the EU AI Act [50], are increasingly moving to prohibit or restrict AI systems that claim to perform “emotion recognition” for the purposes of social control or state surveillance, precisely because the scientific basis for such claims is so fragile. The Walla model provides a clear terminology (vocabulary) that is able to improve any communication about this topic.

### 7.1. Data Dignity and Informed Consent

The use of objective physiological measures (e.g., SRM, EEG, SC) to capture affective processing raises its own set of ethical issues. Because these measures access subcortical, unconscious processes, the user may not even be aware of what they are “revealing”. This challenges the traditional notion of informed consent—how can a person consent to revealing a state that they themselves are not consciously aware of? On one hand, the Walla model emphasizes the behavioral nature of typical data collection and its vague reflection of deep inner states, while on the other hand it raises concerns: should one assume the deep inner state of a person based on external data collection? As a result of that, sole external data collection seems rather acceptable, but if life-affecting decisions are made under the assumption that deep inner states can be accessed, great caution is needed. Due to the above-mentioned diagnostic gap, wrong assumptions can be made, which would certainly be potentially dangerous. There is need for “data dignity” and algorithmic accountability. This includes data minimization, as in collecting only the physiological data necessary for a specific task. When one starts to look into non-conscious information processing in the human brain, there could be ways to get access to information that are not relevant for “emotion recognition”, but are accessible. Such information should be left untouched, while only using affective processing-related information. Also important is ensuring that the “logic” of the emotion recognition model is transparent to the user, allowing them to understand how their biometric features are being used to generate a classification.

### 7.2. Addressing Algorithmic Bias

Algorithmic bias in emotion recognition is often a result of training sets that rely on “universal” facial expressions. By recognizing that expressions (emotions) are social signals, the Walla model acknowledges that they are inherently culturally bound. For example, a “smile” may signal happiness in one culture but serve as a defense mechanism or a signal of politeness in another. AI developers using the Walla framework are encouraged to treat facial data as one part of a broader context. Instead of a one-size-fits-all model, they can build “region-specific fine-tuning” and incorporate demographic data to ensure that the system is not unfairly penalizing individuals whose emotional signaling differs from the “norm” encoded in the training data. This excacerbates the above-mentioned diagnostic gap problem.

## 8. A Roadmap for Next-Gen Affective AI for Defining Specifications

For AI-driven emotion recognition to evolve, this work proposes to integrate the insights of the Walla model into its core architecture. This involves a shift from image analysis to dynamic, multimodal triangulation. Developers are encouraged to stop labeling facial data with internal state words like “Happiness” or “Anger”. Instead, they should label them as behavioral signals—e.g., “Positive Communicative Signal”. This simple change prevents the model from overreaching into the domain of “sentience” or “mind reading” and focuses its accuracy on the actual behavioral output. Whenever possible, AI models should be trained on datasets that pair videos of expressions with synchronous physiological data. By training the AI to recognize the subtle facial patterns that correlate with high-arousal, negative-valence physiological states, we can create systems that are far more sensitive to “genuine” affect than those trained on feigned expressions. The “killer app” for Walla-integrated AI is the detection of incongruence. In security, healthcare, or customer service, the most valuable information is not “what is the user showing?” but “is what they are showing real?”. By measuring the “distance” between the emotion (signal) and the affective processing (source), AI can identify instances of deception, social masking, or repressed distress that would be invisible to current unimodal systems. Traditional AI models typically rely on facial expression analysis [51]. However, social masking is specifically the act of decoupling this outward expression from the internal state [52]. Only by measuring physiological data can the biological truth of an individual’s reaction be detected even when their facial expression is perfectly masked to appear neutral or positive. AI-driven systems might mistake a masked smile for genuine happiness, because they lack the capacity to see the underlying feeling or even more so the raw affective processing. In cases where social masking is a survival mechanism (e.g., in neurodivergent individuals), the integration of objective neurophysiological markers is essential. This allows for the detection of high-stress levels or negative valence that an AI would otherwise overlook, ensuring that the individual’s actual inner state is understood rather than just their performative emotion.

In the future, we will see an increase in available “neuro-adaptive” systems that are meant to optimize human–machine interaction. These systems would monitor a user’s affective processing (via non-invasive sensors like a Smart Watch or an EEG headband) to detect technostress or frustration before the user even realizes it. The system could then adjust the interface, offer a break, or change the difficulty of a task to maintain the user’s optimal affective state. This will only work if raw affective data are included. A fundamental shift from “Emotion Recognition” (reading faces) to “Affection Recognition” (reading the raw neural signal) is needed. Relying on “synthetic emotions”—facial expressions or vocal tones that humans consciously perform—is considered dangerous, because these are just communicative outputs, often disconnected from what the person actually feels.

Most importantly, it is recommended to accept that affective processing, feelings and emotions are basically three different and separate streams of information. For instance, if the internal feeling (anxiety) does not match the external emotion (a smile), the AI identifies “Affective Dissonance”, a key metric for mental health, leadership, and high-stakes decision-making. The AI model must learn to treat feelings (subjective experience), emotions (behavioral expression) and raw affective processing as three separate data streams.

## 9. Synthesis and Conclusions

In this theoretical paper, a potentially helpful paradigm shift for the field of affective computing and emotion recognition in particular is proposed. By rigorously distinguishing between the three discussed concepts, a potential solution to the conceptual confusion that has also plagued emotion research itself for decades is offered with high relevance for AI. Its utility for AI extends far beyond a simple vocabulary fix. Its most dominant argument is rooted in the functional separation of signal from source, which allows for the detection of incongruent scenarios by identifying discrepancies between shown expressions and felt states, a critical requirement for deception detection and social intelligence. Another important aspect is the mitigation of cognitive pollution via bypassing the biases of self-report and language to access the “raw” evaluative data of the brain. Providing a scientific basis for differentiating between the public signaling of emotion and the private experience of feeling, essential for creating robust privacy and regulatory frameworks, should give ethical clarity. Finally, offering objective physiological markers (e.g., SRM, EEG, SC) can serve as a more reliable “ground truth” for training AI than subjective human labels.

As AI becomes more integrated into our social and professional lives, the ability to understand and respect the biological reality of human affect will be paramount. The Walla model provides the neurobiological blueprint for the generation of an AI model that is not just technically sophisticated, but humanly aware, and able to navigate the complex “diagnostic gap” between what we show the world and what our brains actually know. By grounding AI in the hierarchical and evolutionary logic of the brain, we can move toward affective computing that is more accurate, more ethical, and ultimately more helpful to the humans it serves.

## Figures and Tables

**Table 1:** The Walla Emotion Model: Key concepts and AI benefits.

Phenomenon	Definition	Biological Basis	AI Benefit
Affective processing	Non-conscious, automatic, neurophysiological stimulus evaluation coding (good or bad?).	Subcortical limbic system neural activity.	Raw evaluations before potential masking; affective truth underlying decision-making.
Feeling	Conscious interpretation of affective state.	Limbic system activity causes release of chemicals, which is felt.	Verbally reported feeling might be different to actual affective truth.
Emotion	External, communicative behavioral signal.	Motor activity causing various muscles to contract.	Identify potential mismatch between external signal and affective truth.

**Table 2:** Implicit and explicit measures of human responses.

Measure Type	Example	Access Point	Potential for Bias
Explicit	Surveys, Interviews	Conscious reasoning	High (Cognitive Pollution)
Implicit	EEG, ERPs SRM, SC	Neural/Physiological activity	Low (Direct measurement)

## Data Availability

No new data were created or analyzed in this study. Data sharing is not applicable to this article.
